# Nucleic acid-induced chemokine expression in keratinocytes: Implications for skin inflammation

**DOI:** 10.1371/journal.pone.0336901

**Published:** 2025-11-20

**Authors:** Judit Danis, Evelyn Kelemen, Fanni Balogh, Kornélia Szabó, Gergely H. Fodor, Éva Ádám, Márta Széll

**Affiliations:** 1 Department of Immunology, Albert Szent-Györgyi Medical School, University of Szeged, Szeged, Hungary; 2 HUN-REN-SZTE Dermatological Research Group, University of Szeged, Szeged, Hungary; 3 Department of Dermatology and Allergology, Albert Szent-Györgyi Medical School, University of Szeged, Szeged, Hungary; 4 HCEMM-SZTE Skin Research Group, University of Szeged, Szeged, Hungary; 5 Department of Medical Physics and Informatics, Albert Szent-Györgyi Medical School, University of Szeged, Szeged, Hungary; 6 Department of Medical Genetics, Albert Szent-Györgyi Medical School, University of Szeged, Szeged, Hungary; 7 HUN-REN-SZTE Functional Clinical Genetics Research Group, University of Szeged, Szeged, Hungary; Arizona State University, UNITED STATES OF AMERICA

## Abstract

Chemokines play an important role in the pathogenesis of skin diseases, such as psoriasis, atopic dermatitis, vitiligo, and alopecia areata. Recently literature data supports the theory that alternatively spliced isoforms of these molecules may serve as potential regulators in these diseases. Since self-derived nucleic acids are main culprits in chronic skin diseases we compared the effects of synthetic RNA- and DNA-induced inflammation on the expression levels of chemokines in human keratinocytes. We found that cytoplasmic nucleic acids are potent inducers of monocyte chemoattractant protein-1 (CCL2), interferon gamma inducible protein-10 (CXCL10) and fractalkine (CX3CL1) mRNA-expression, mainly through NF-κB activation, but the pattern recognition receptors responsible for inducing this activation are still unknown. Alternative splicing of these chemokines in keratinocytes was not detected, suggesting other regulatory mechanisms for chemokine activity.

## Introduction

The skin serves as the primary barrier against environmental threats, with keratinocytes playing a crucial role in both physical protection and immune response. As immunocompetent cells, keratinocytes produce various mediators, including chemokines, acting in the recruitment and activation of immune cells. Chemokines are notably overexpressed in chronic skin diseases [1] such as psoriasis [2–4], atopic dermatitis [5], alopecia areata [6], vitiligo [7]. Their expression can be triggered by cytokines and microbial products via pattern recognition receptors (PRRs) [8].

Keratinocytes express several classes of PRRs that detect pathogen-associated and damage-associated molecular patterns (PAMPs and DAMPs), enabling them to respond to infectious or sterile inflammatory cues [9–12]. Among these stimuli, RNA and DNA fragments can function as both PAMPs and DAMPs, playing a protective role in antiviral immunity [13,14] but also contributing to chronic inflammation when dysregulated [15–19]. Inflammatory skin conditions are frequently characterized by the accumulation of self-derived nucleic acids in skin and serum [16], which can stem from defective nuclease activity [20,21], mitochondrial disfunction [22,23] or excessive neutrophil extracellular trap (NET) formation [24].

These extracellular or cytosolic nucleic acids are potent activators of keratinocyte immune responses, including type I interferon (IFN-α/β) and inflammatory cytokine expression [15,25,26] and inflammasome activation [16,27]. However, while cytokine responses to nucleic acids in keratinocytes have been extensively characterized, their impact on chemokine expression remains poorly defined. This represents a clinically relevant knowledge gap, as chemokines are central regulator of immune cell recruitment, shaping disease initiation and progression, and therapeutic responsiveness in inflammatory skin conditions [28,29].

To address this knowledge gap, we applied a focused experimental design. First, we performed a targeted screen of nucleic acid-induced chemokine gene expression in keratinocytes [30]. Based on these results, we conducted mechanistic analysis of the most significantly induced candidates.

Three chemokine families [31] were included in the screening approach: (1) CC chemokines, which primarily mediate monocyte and macrophage recruitment and are frequently elevated in chronic inflammatory skin diseases [4]; (2) CXC chemokines, which are often interferon-inducible [32] and serve as critical mediators linking innate pathogen sensing with T cell and NK cell recruitment [5,7,33]; and (3) CX3C chemokines, which represent a unique subfamily with dual adhesion and chemotactic functions, playing specialized roles in leukocyte positioning and tissue surveillance [2,3,34].

In addition, recent evidence suggests that chemokine activity can be significantly modulated through post-transcriptional mechanisms such as alternative splicing, generating isoforms with altered receptor specificity, stability, or functional activity [35,36]. To understand, whether this mechanism contributes to chemokine regulation in keratinocytes, we explored the presence of splice isoforms both in public datasets and following nucleic acid stimulation.

By focusing on a defined set of nucleic acid-induced chemokines, their expression regulation and potential splice isoforms, our study provides mechanistic insight on how keratinocytes convert nucleic acid sensing into immune cell recruitment signals to shape chronic skin inflammation.

## Materials and methods

### Cell culture

Third passage primary human epidermal keratinocytes (NHEKs) were used in the experiments. Skin specimens were obtained from aesthetic breast surgery of women (average age 44.05 ± 10.09 years) performed between 01/03/2020-30/11/2023 at the Plastic Surgery Unit of the Department of Dermatology and Allergology of the University of Szeged, and were used to separate NHEKs. The epidermis was separated from the dermis with overnight incubation in Dispase (Roche Diagnostics, Manheim, Germany), and keratinocytes were obtained after maceration in 0.25% trypsin. The NHEKs were grown in 75 cm^2^ cell culture flasks for two passages in serum-free keratinocyte medium containing epidermal growth factor and bovine pituitary factor (Gibco Keratinocyte SFM Kit; Life Technologies Kft., Budapest, Hungary) and supplemented with 1% antibiotic/antimycotic solution (Capricorn Scientific, Ebsdorfergrund, Germany) and 1% L-glutamine (Capricorn Scientific) at 37 °C in a humidified atmosphere with 5% CO_2_. The medium was changed every two days.

The investigations were carried out according to the rules of the Declaration of Helsinki, and prior study, the study design was approved by the Human Investigation Review Board of the University of Szeged (HCEMM-001). Written informed consent was obtained from each investigated individual.

### Experimental procedures

Third-passage NHEKs were seeded into 6-well plates at a density of 200 000 cells/ml in complete medium. After 24 hours, cells were transfected with 1 μg/mL polydeoxyadenylic acid-polydeoxythymidylic acid double-stranded homopolymer (poly(dA:dT)) (InvivoGene, San Diego, CA, USA) or with 0.666 μg/mL polyinosinic-polycytidylic acid (poly(I:C)) (Sigma Aldrich, Saint Louis, MO, USA) using the X-tremeGene 9 transfection reagent (Roche Diagnostics). For some experiments, cells were treated with 100 μg/mL IL-17A, 100 μg/mL IL-23, 100 μg/mL IL-12, 50 ng/mL TNFα, 1 μg/mL IMQ, 40 nM PMA, 500 ng/ml LPS or live *Cutibacterium acnes* (*C. acnes*) strain 889 using at a multiplicity of infection (MOI) of 1:100. Cells were harvested at the indicated time points.

Nucleic acid sensing receptor silencing was carried out using siRNA-mediated gene silencing with the ON-TARGETplus SMARTpool for RIG-I, IFIH1, TLR3, cGAS, ZBP1, IFI16, NLRP1, NLRP3, AIM2 siRNAs, or as control the ON-TARGETplus nontargeting pool (Dharmacon, Lafayette, CO, USA) at a final concentration of 40 nM. Transfection was carried out using the X-tremeGENE siRNA transfection reagent (Roche Diagnostics), according to the manufacturer’s instructions. Effectivity of silencing was determined by qPCR.

For inhibition studies, cells were incubated for 60 minutes prior to poly(dA:dT)/poly(I:C) transfection with inhibitors for NF-κB (Bay 11−7085, 10 μM; MedChem Express, Monmouth Junction, NJ, USA), STING (H151, 200 ng/ml, InvivoGene) STAT-1 (Fludarabine, 10 μM; Sigma Aldrich), STAT-3 (Stattic, 5 μM; Sigma Aldrich), MEK1 (PD98059, 20 μM; Sigma Aldrich), JNK (SP600125, 10 μM; Tocris Bioscience, Bristol, UK) and p38 (SB203580, 10 μM; Tocris Bioscience).

### RNA isolation, qPCR array and real-time RT-PCR

Cells were harvested in TRI Reagent^®^ (Zymo Research Corporation, Irvine, CA) at the indicated time points and total RNA was isolated according to the manufacturer’s instructions. The potential contamination of genomic DNA was removed using the Turbo DNA-free Kit (Invitrogen, Life Technologies) according to the manufacturer’s instructions. 1 µg of total RNA was reverse transcribed into cDNA by the Ultrascript 2.0 cDNA Synthesis Kit (PCR Biosystems Ltd., London, UK).

Samples for the qPCR array were harvested 6 hours after transfection in TRI Reagent ^®^. RNA isolation was carried out using the Direct-Zol RNA miniprep kit (Zymo Research), genomic DNA contamination was removed using the Turbo DNA-free Kit (Invitrogen, Life Technologies). cDNA was synthesized using the RT^2^ first-strand cDNA synthesis kit (Quiagen). An RT^2^ Profiler PCR array (Quiagen) was performed using the Luminaris SYBR-Green mix (Life Technologies), the custom gene list has been previously described [30].

Real-time RT-PCR experiments were performed with the TaqMan^TM^ gene expression assay (Life Technologies) and the Lo-ROX qPCRBIO Probe Mix (PCR Biosystem) on a C1000 Touch Thermal Cycler (Bio-Rad Laboratories). The expression of each gene was normalised to the expression of the GAPDH gene. Relative mRNA levels were calculated using the ΔΔ*C*_t_ method compared to the nontreated control samples.

### Determination of splice-variants by PCR

To determine splice variants of fractalkine, PCR reactions were carried out using the DreamTaq Green DNA Polymerase (Thermo Fischer Scientific) according to the manufacturer’s instructions. Primers were designed to overlap splice-sites, to allow for specific amplification of splice-variants NM_002996.4 and NM_001304392.1. Primers are listed in S1 Table. PCR products were run on 4% agarose gel and products were cut out and purified by NucleoSpin Gel and PCR Purification Kit (Macherey-Nagel) and subjected to Sanger sequencing.

### Statistical Analysis

Gene expression differences were analyzed using linear mixed-effects models with donor as a random effect to account for inter-individual variability, implemented in R (version 4.2.1) using the lme4 package. Gene expressions were log2-transformed prior to analysis.

For the inhibitor experiments, each inhibitor was compared to its corresponding solvent control (DMSO250, DMSO1000, or DMSO2500) within each stimulus condition (poly(I:C) and poly(dA:dT)). For the silencing experiments, gene expression upon each siRNA transfection was compared to the non-targeting control siRNA transfection within each stimulus condition. For the stimulus response experiments, the effects of different stimulation were compared to unstimulated control. For the assessment of time kinetics, expressions were compared to baseline (0 hours) data.

Pairwise comparisons were performed using the emmeans package. For the inhibitor and silencing experiments, p-values were adjusted using the Benjamini-Hochberg method within each solvent group-stimulus combination (inhibitor experiments) or within each stimulus condition (silencing experiments). For the stimulus response experiments, comparisons of each stimulus versus control were performed with Holm adjustment. Statistical significance was defined as adjusted p < 0.05.

## Results

### Cytosolic nucleic acids induce the expression of chemokines in human keratinocytes

**Fig 1:**
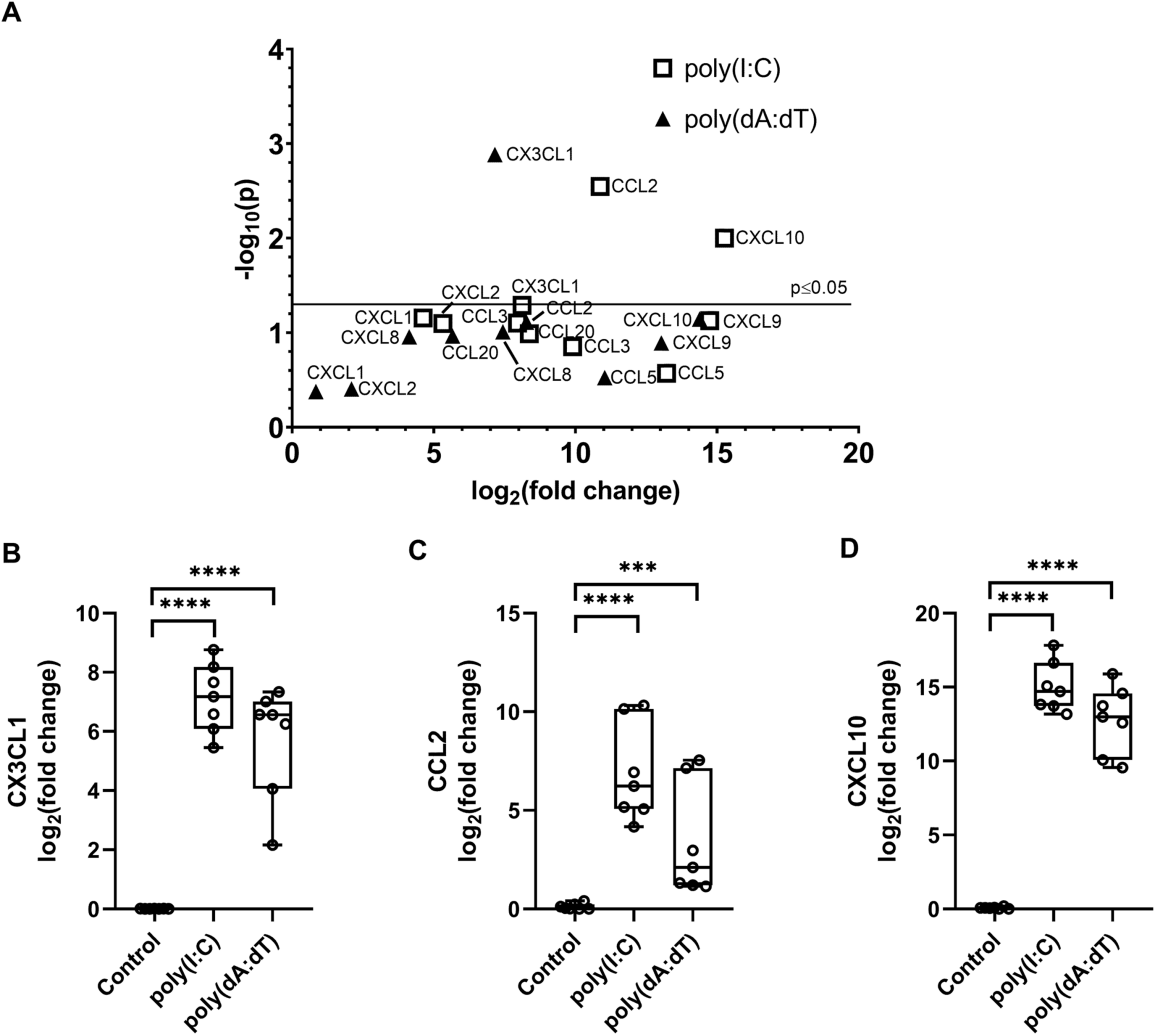
Expression of chemokines upon RNA – poly(I:C) or DNA – poly(dA:dT) transfection. **(A)** Results of a qPCR array on chemokines, carried out on keratinocyte samples transfected by poly(I:C) (white square) or poly(dA:dT) (black triangle) for 6 hours. Expression changes were compared to mock transfected samples, results are presented as volcano plots, significant changes (p < 0.05) are displayed above the horizontal line. Experiments were carried out on cells from four independent donors, statistical analysis used linear mixed-effects models with donor as a random effect to account for inter-individual variability.

In a previous qPCR-array [30], we identified genes that responded with altered expression to transfection with the synthetic nucleic acid analogues poly(I:C) or poly(dA:dT) in NHEKs. The array included various cytokines, chemokines, antimicrobial peptides, and cytoplasmic receptors, which appear to be key players in psoriatic skin cells [30]. Among the genes studied, 10 chemokines were included (CCL2, CCL3, CCL5, CCL20, CXCL1, CXCL2, CXCL8, CXCL9, CXCL10, CX3CL1), all known to originate from keratinocytes and play important roles in chronic skin diseases [2–7,37]. We observed significantly elevated expression of CCL2 (Monocyte chemoattractant protein-1, MCP-1) and CXCL10 (Interferon gamma inducible protein-10, IP-10) after poly(I:C) transfection while fractalkine (CX3CL1) expression was significantly increased following poly(dA:dT) transfection (Fig 1A). These three chemokines have been implicated in the pathogenesis of skin diseases [2,5,7], particularly by recruiting professional immune cells to the site of injury or infection. The qPCR array results were validated using keratinocytes from seven independent donors, confirming the upregulation of these genes upon synthetic RNA or DNA transfection (Fig 1B-D). The kinetics of their expression was studied for 48 hours posttransfection, showing varying degrees and kinetics of activation between the three chemokines. Particularly CCL2 and fractalkine were rapidly induced to a similar level (S1 Fig), CXCL10 exhibited 5–6-times higher induction in expression, and this expression stayed more stable for longer(S1 Fig).

B-D) Validation of the qPCR array on independent donors for fractalkine (B), CCL2 (C), and CXCL10 (D) showing mRNA expression in NHEKs upon nucleic acid induction. Cells were transfected with 0,666 μg/mL poly(I:C) (white squares) and 1 μg/mL poly(dA:dT) (black triangles), and samples were collected at 6 hours after transfection. Relative mRNA expression was determined by the ∆∆Ct method, GAPDH was used as normalizing gene, fold changes were calculated compared to the expression of the untreated (Control) samples. Data are presented as box plots with individual datapoints included, n = 7. Gene expression differences were analyzed using linear mixed-effects models, comparisons of each poly(I:C) or poly(dA:dT) stimulus versus control were performed with Holm adjustment ***p < 0.001, ****p < 0.0001.

### Chemokine gene expression is predominantly induced by nucleic acids in keratinocytes

**Fig 2:**
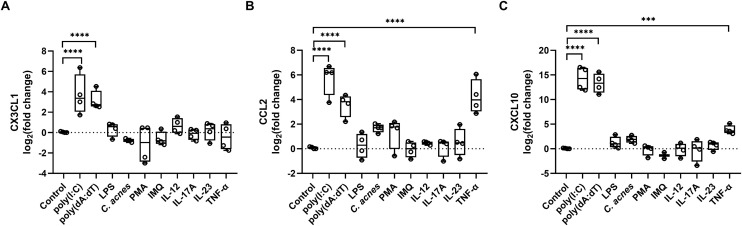
Expression of chemokines fractalkine (A), CCL2 (B) andd CXCL10 (C) upon different inflammatory stimuli. Cells were transfected with 0,666 μg/mL poly(I:C), 1 μg/mL poly(dA:dT) or treated by 100 ng/mL IL-17A, 100 ng/mL IL-23, 100 ng/mL IL-12, 50 ng/mL TNFα and 1 μg/mL IMQ, 40 nM PMA, 500 ng/ml LPS or 1:100 MOI live *Cutibacterium acnes (C. acnes)* strain 889 bacteria. Cells were harvested 24 hours post treatment. Relative mRNA expression was determined by the ∆∆Ct method, using GAPDH as a normalizing gene and fold changes were calculated compared to the expression of the untreated control samples. Data are presented as box plots, including individual datapoints, n = 4. Gene expression differences were analyzed using linear mixed-effects models, comparisons of each stimulus versus control were performed with Holm adjustment ***p < 0.001, ****p < 0.0001.

In the skin, keratinocytes encounter various PAMPs and DAMPs. To mimic these conditions, we treated keratinocytes with several cytokines and microbial compounds associated with psoriasis. In contrast to poly(I:C) and poly(dA:dT), which were able to induce significant upregulation in all three studied chemokines, treatment with lipopolysaccharide (LPS), a component of Gram-negative bacteria, *Cutibacterium acnes* (*C. acnes*), imiquimod and phorbol-myristate-acetate (PMA) did not induce their expression (Fig 2). Psoriasis-specific cytokines (e.g., IL-12, IL-17A, IL-23) did not promote chemokine expression, but TNF-α – which could indicate both infection and sterile inflammation – was able to induce CCL-2 and CXCL10 expression (Fig 2B,C). Interestingly, fractalkine expression was predominantly induced by nucleic acids with no induction observed from the cytokines tested, suggesting its expression is specifically regulated. (Fig 2A).

### Nucleic acid induced expression of chemokines is mediated through NF-κB signaling

Since the expression of the three studied chemokines – CCL2, CXCL10 and fractalkine – was primarily induced by nucleic acids, it raises two questions: which nucleic acid receptors are activated and which signaling pathways are involved in these processes. We studied nine nucleic acid sensing receptors – RIG-I, IFIH1, TLR3, cGAS, ZBP1, IFI16, NLRP1, NLRP3 and AIM2 – whose role has already been described in nucleic acid-induced immune reactions in various cell types [14,16,38–42]. However, silencing of these receptors did not alter the expression of any of the three studied cytokines (S2 and S3 Fig).

**Fig 3:**
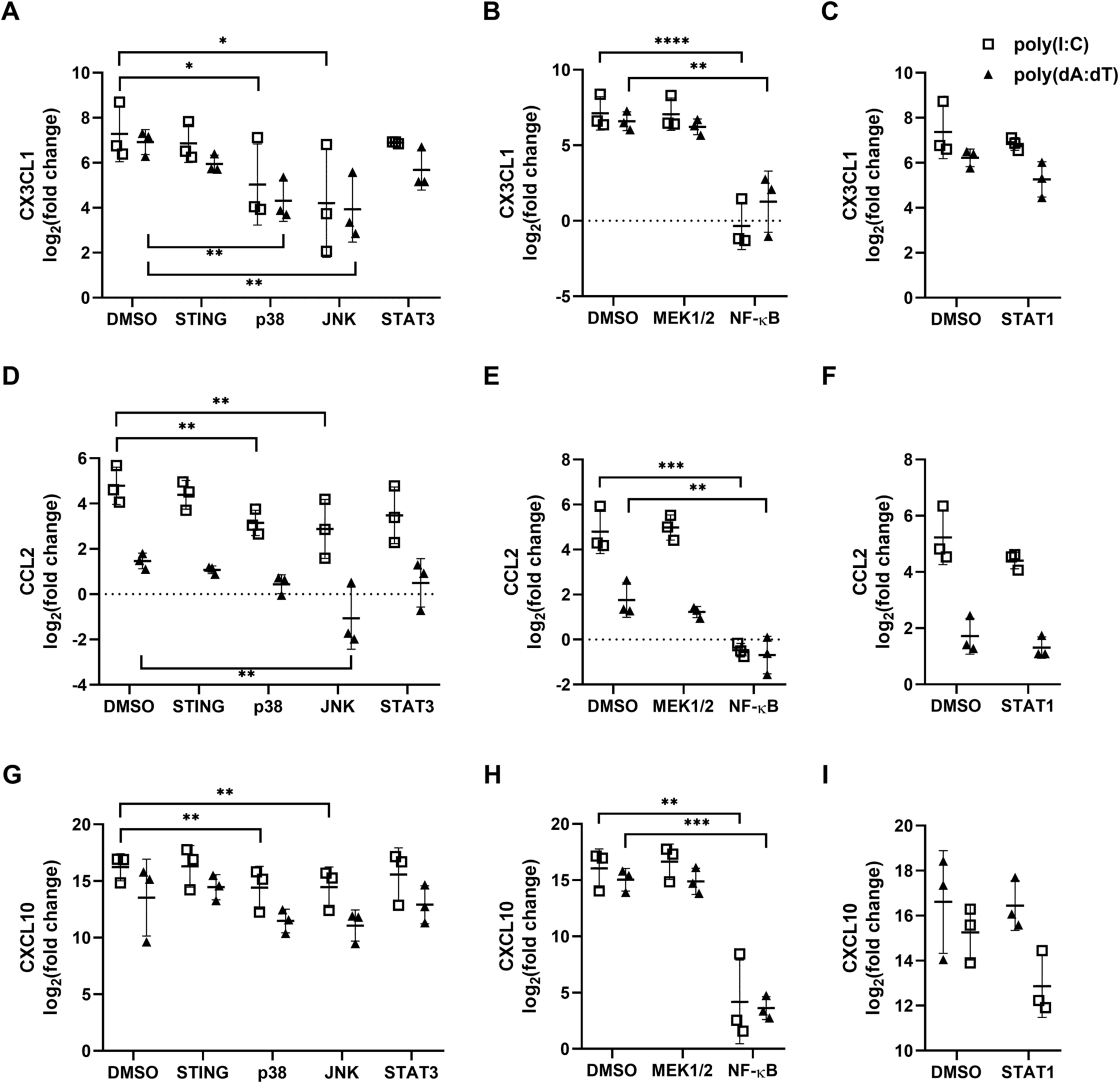
Inhibition of the NF-κB, p38 and JNK pathway components had an effect on the nucleic acid induced chemokine expression of NHEKs. Specific inhibiton of signaling pathway components was obtained by inhibitors (NF-κB (Bay 11−7085, 10 μM); STING (H151, 200 ng/ml); STAT-1 (Fludarabine, 10 μM); STAT-3 (Stattic, 5 μM); MEK1 (PD98059, 20 μM); JNK (SP600125, 10 μM) and p38 (SB203580, 10 μM)) for 60 minutes before transfecting the cells with 0,666 μg/mL poly(I:C) or 1 ug/mL poly(dA:dT). Gene expression of fractalkine (A-C), CCL2 (D-F) and CXCL10 (G-I) was determined by the ∆∆Ct method, with GAPDH as a normalizing gene compared to the expression of the nontreated control samples. Data are presented as mean ± SD with individual datapoints, white squares representing poly(I:C), black triangles representing poly(dA:dT) treatment, n = 3. Gene expression differences were analyzed using linear mixed-effects models, p-values were adjusted using the Benjamini-Hochberg method within each solvent group-stimulus combination **** p < 0.0001, *** p < 0.001,** p < 0.01, *p < 0.05.

Previously, we have found that NF-κB, MAPK and STAT pathways are involved in nucleic-acid-induced responses in keratinocytes [43]. By applying specific inhibitors, the involvement of the NF-κB, MAPKs, STAT and STING pathways in chemokine expression was tested. Among them, inhibition of NF-κB diminished chemokine expression, while p38 and JNK MAP kinase pathways appeared to have a minor role in poly(I:C) or poly(dA:dT)-induced chemokine expression (Fig 3).

Based on these results, we concluded that nucleic acid-induced NF-κB activation is the main pathway leading to chemokine expression; however, it is not yet clear which nucleic acid-sensing receptors are responsible for the activation of this pathway.

### The role of alternative splicing in the regulation of chemokine expression

Alternative splicing of inflammatory mediators can alter their functions [44–46] and the role of alternative splicing as a regulatory mechanism in keratinocytes has already been established [47–49]. Based on these, we were interested in whether alternatively spliced mRNA isoforms of the studied chemokines are detectable in keratinocytes upon nucleic acid stimuli.

Using the NCBI database [50], we identified fractalkine as a chemokine having two protein-coding isoforms, while CCL2 and CXCL10 do not have alternative splice variants in the database. For fractalkine, two isoforms are shown: the full-length variant (NM_002996.6) and a shorter one, lacking the 2nd exon (NM_001304392.3) (S4 Fig). In the latter, the chemokine motif of the protein is missing, so the isotype probably does not have chemotactic activity. To identify the two variants in keratinocytes, various pairs of PCR primers were used (S1 Table), but only the full-length variant could be confirmed in our experiments, which was verified by sequencing (S5 Fig).

According to the ENSEMBL database [51], the shorter variant of fractalkine (ENST00000564948.1) is predicted as TSL3, in which “splice junctions are supported by only one EST”, while the full-length variant (ENST00000006053.7 identical to NM_002996.6) is of high certainty (TSL1: all splice junctions of the transcript are supported by at least one non-suspect mRNA). In the USCS Genome Browser, only one EST represented the shorter splice variant (CA396869) [52], which was found in human choroid in the eye [53].

Additionally, we also analyzed several publicly available RNA-Seq datasets to investigate whether cell types highly expressing fractalkine would express both splice variants, but in human airway epithelial cells [54], differentiated human keratinocytes [55] and in human nasopharyngeal carcinoma cells [56], where fractalkine is significantly up-regulated, we also could not confirm the expression of the shorter transcript variant.

## Discussion

Chemokines are critical regulators of cutaneous immunity shaping both physiological host defence and pathological inflammation [2–7,37]. In this study, we demonstrate that cytosolic nucleic acids are potent inducers of selected chemokines in human keratinocytes, namely CCL2, CXCL10 and fractalkine. These three identified chemokines are particularly significant as they represent functionally distinct classes that collectively orchestrate key inflammatory processes in skin immunity and provide a comprehensive framework for understanding how nucleic acid stimulation shapes distinct immune cell recruitment and activation pathways.

CCL2 (MCP-1), a chemokine of the C-C subfamily, is significantly elevated in psoriasis and atopic dermatitis, contributing to immune cell recruitment and antiviral defense [5]. CCL2 primarily recruits monocytes and macrophages and is consistently elevated in multiple inflammatory skin diseases including psoriasis and atopic dermatitis [5].

CXCL10 (IP-10) is a type I interferon-inducible chemokine that attracts activated T cells and NK cells, serving as a critical molecular link between innate nucleic acid sensing pathways and adaptive immune activation [5,7,33]. It is upregulated in vitiligo and psoriasis [5,7].

Fractalkine, uniquely functions both as a membrane-bound adhesion molecule and soluble chemoattractant, playing essential roles in regulating dendritic cell and T cell positioning within inflamed skin tissues [3,35] particularly in psoriatic lesions [2]. Moreover, there is an interplay between CCL2 and fractalkine, and CCL2 can compensate for loss of fractalkine during macrophage recruitment [3].

Our work confirms and extends prior reports, that synthetic nucleic acids act as danger signals in skin [14–17]. Notably, poly(I:C) elicited faster and more pronounced expression compared to poly(dA:dT), suggesting a differential mechanism of action. Despite the established roles of cytokines, such as TNF-α, IFN-γ and IL-17 in the induction of these chemokines [2,5], our results highlight that the presence of nucleic acid fragments is a potent stimulus for chemokine expression [37].

Interestingly, silencing key nucleic acid receptors [14,38,39,57], including TLR3, MDA5, RIG-I, IFI16 and cGAS, did not affect chemokine expression. Some inflammasome sensors were also shown to be responsible for the detection and response to nucleic acids in keratinocytes [16,40–42], but their silencing did not have an effect on the expression of chemokines. These results indicate that alternative receptors may mediate this response in keratinocytes.

Nucleic acid sensing receptors induce different signaling pathways including MAP kinases, STAT, STING, and NF-κB activation [26,38,43,58–60]. The inhibition of p38, JNK, and NF-κB pathways resulted in decreased chemokine expression, highlighting the necessity of these pathways in the inflammatory response, while STAT or cGAS-STING pathways were not affecting chemokine expression in keratinocytes.

Our results show that the complex interplay of signaling pathways is particularly critical for chemokine transcription. All studied chemokines harbor multiple NF-κB promoter elements upstream of their transcription start site, and lower number of activator protein 1 (AP1) elements [32,61] which likely contributes to the differential effects of NF- κB, p38 and JNK on chemokine expression. The fractalkine promoter has numerous putative binding sites for NF-κB, CCAAT-enhancer binding protein (C/EBP), activator protein-1 (AP-1) and STAT transcription factors, of which NF-κB induces maximal promoter activity [62]. Similarly, we found that NF-κB inhibition completely diminished the expression of all three chemokines, while the inhibition of other pathways had less effect. It is in line with findings, that the additional promoter elements are only turned on when the NF-κB binding elements are non-functional [62]. However, our study of selected signaling cascades is a limited approach, and although provides valuable mechanistic insights, it does not exclude the potential involvement of other regulatory networks that were not investigated in this study, and cannot fully recapitulate the complexity of endogenous pathway regulation.

Alternative splicing is a key factor in increasing cellular and functional complexity [36]. Various databases described alternative splicing of fractalkine mRNA (S4 Fig), suggesting multiple levels of regulation of fractalkine function, since it is known to undergo post-translational modification to form a membrane-bound and soluble form [35]. However, despite these claims on alternative splicing, our investigations have not revealed distinct splice variants for fractalkine in keratinocytes. This observation, together with RNA-Seq data from multiple cell types and UCSC Genome Browser data showing only one EST for the shorter splice variant [52], raises questions about the validity of the reported alternative splicing events. It appears that this isoform may only be expressed under very specific conditions or cell types and is probably not present in most human cells. Furthermore, the absence of any suspected fractalkine splice variants in mouse models further supports this notion [63].

### Clinical and therapeutic implications

Our results demonstrate the critical role of nucleic acids in driving chemokine expression in keratinocytes, and highlight the need for further investigation into the receptors and pathways involved. Rather than solely focusing on downstream effector molecules such as IL-17 and IL-23 [64], our findings indicate that targeting upstream nucleic acid sensing or NF-κB/MAPK signaling pathways may provide broader anti-inflammatory effects. Understanding the activation and role of chemokines in keratinocytes could lead to the development of adjunct therapies that specifically modulate chemokine signaling, thereby enhancing the efficacy of existing treatments. Moreover, targeting the receptors or downstream signaling pathways involved in nucleic acid sensing may offer a new avenue for intervention.

### Study limitations and technical considerations

Our study employed primary human keratinocytes, which inherently exhibit substantial biological variability between donors, reflecting the natural diversity of human immune responses. While this variability resulted in large error bars in some experiments, the consistent directional changes across all donors demonstrate robust and reproducible effects. This biological variability, rather than representing a weakness, actually strengthens the clinical relevance of our findings by demonstrating that nucleic acid-induced chemokine responses are consistent across genetically diverse human populations.

The scope of our methodological approach was necessarily focused to enable comprehensive mechanistic analysis within practical constraints. Our qPCR array screening provided broad chemokine profiling, while targeted qPCR analysis allowed detailed characterization of the most significantly responding candidates. While more comprehensive approaches such as RNA-sequencing or proteomics could provide additional insights, our targeted strategy enabled thorough investigation of key pathways while maintaining experimental feasibility with primary cell systems that have limited expansion capacity.

## Conclusions

Our research underscores the critical role of nucleic acids in driving chemokine expression in keratinocytes through NF-κB and MAPK-dependent pathways, despite apparent redundancy in upstream sensing mechanisms. While our focused methodological approach and the inherent variability of primary human cells present certain limitations, these findings provide strong rationale for the clinical relevance of nucleic acid induced chemokine signaling in inflammatory skin diseases. From a translational perspective, defining these mechanisms could open avenues for innovative therapeutic strategies aimed at modulating chemokine signaling and improving outcomes for patients with inflammatory skin diseases.

Finally, our analyses of multiple RNA-seq datasets and our experimental data did not reveal evidence of fractalkine splicing in keratinocytes, despite database annotations reporting splice variants. This discrepancy underscores the importance for a critical re-evaluation of existing databases and emphasizes that experimental validation is essential before accepting annotations of complex regulatory mechanisms, such as alternative splicing. Updating these resources to reflect current evidence will strengthen the reliability of chemokine biology and prevent misinterpretation in future studies.

## Supporting information

S1 Fig Kinetics of fractalkine (A), CCL2 (B), and CXCL10 (C) mRNA expression in NHEKs upon nucleic acid induction. Cells were transfected with 0,666 μg/mL poly(I:C) and 1 μg/mL poly(dA:dT), and samples were collected at 6, 12, 24, 48 hours after transfection. Relative chemokine mRNA expression was determined by the ∆∆Ct method, normalized to GAPDH mRNA expression and fold changes were calculated compared to the expression of the untreated (Control) 0 hours samples. Data are presented as mean of three independent experiments ± SD, white squares representing poly(I:C), black triangles representing poly(dA:dT) treatment. Gene expression differences were analyzed using linear mixed-effects models, for the assessment of time kinetics, expressions were compared to baseline (0 hours) data, *p < 0.05, **p < 0.01, ***p < 0.001, ****p < 0.0001.
(PDF)

S2 Fig Effectivity of silencing on nucleic acid sensing receptors. Genexpression of each nucleic acid receptor was silenced using siRNA-mediated gene silencing. Relative mRNA expression of each silenced receptor RIG-I (A), IFIH1 (B), TLR3 (C), cGAS (D), ZBP1 (E), IFI16 (F), AIM2 (G), NLRP1 (H), NLRP3 (I) was determined by the ∆∆Ct method, normalized to GAPDH mRNA expression and compared to the expression of the mock-transfected control samples. Data are presented as mean ± SD with individual datapoints, white squares representing poly(I:C), black triangles representing poly(dA:dT) treatment, n = 3. Gene expression differences were analyzed using linear mixed-effects models with donor as a random effect to account for inter-individual variability, each siRNA was compared to the non-targeting control siRNA within each stimulus condition, * p < 0.05, **p < 0.01.
(PDF)

S3 Fig Silencing of none of the nucleic acid sensing receptors interferes with chemokine expression. Expression of pattern recognition receptors were silenced with siRNA mediated inhibiton and the cells were transfected with 0,666 μg/mL poly(I:C) and 1 μg/mL poly(dA:dT). Relative fractalkine (A), CCL2 (B) and CXCL10 (C) mRNA expression was determined by the ∆∆Ct method, normalized to GAPDH mRNA expression and compared to the expression of the mock-transfected control samples. Data are presented as mean ± SD with individual datapoints, white squares representing poly(I:C), black triangles representing poly(dA:dT) treatment, n = 3. Gene expression differences were analyzed using linear mixed-effects models with donor as a random effect to account for inter-individual variability, each siRNA was compared to the non-targeting control siRNA within each stimulus condition.
(PDF)

S4 Fig NCBI database records of fractalkine, CCL2 and CXCL10 chemokines. Accessed on 10.10.2023.
(PDF)

S5 Fig PCR reactions identified only the NM_002996 variant of fractalkine. To determine splice variant of fractalkine cDNA from keratinocytes transfected by poly(I:C) or poly(dA:dT) was used in PCR reactions using the DreamTaq Green DNA Polymerase (Thermo Fischer Scientific) according to the manufacturer’s instructions. PCR products underwent electrophoresis on 4% agarose gel, and were visualized on the Omega Lum G Chemidoc Imaging System (Aplegen, Inc, Pleasanton, CA, USA). Numbers correspond to the respectieve PCR-primer pairs listed in S1 Table. Products were cut out, isolated by the Nucleospin Gel and PCR Cleanup kit (Macherey Nagel) and subjected to Sanger sequencing.
(PDF)

S1 Table Primer pairs and expected PCR-product sizes for identifying fractalkine splice variants. (PDF)
