# Supplementary figures and images for: Nucleic acid-induced chemokine expression in keratinocytes: Implications for skin inflammation
Source: PLoS One. 2025 Nov 20;20(11):e0336901. doi: 10.1371/journal.pone.0336901 (PMC12633943; doi:10.1371/journal.pone.0336901)

**A**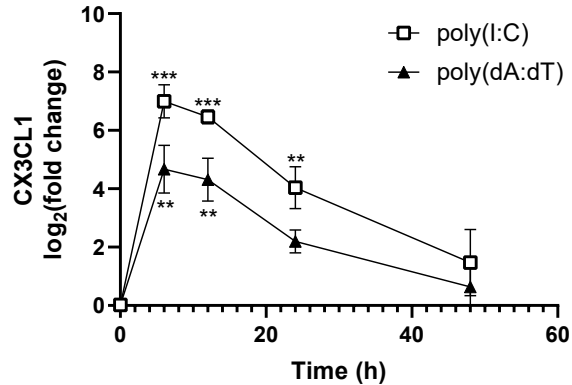**B**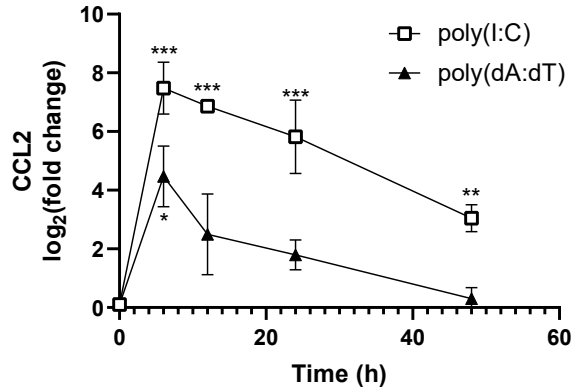**C**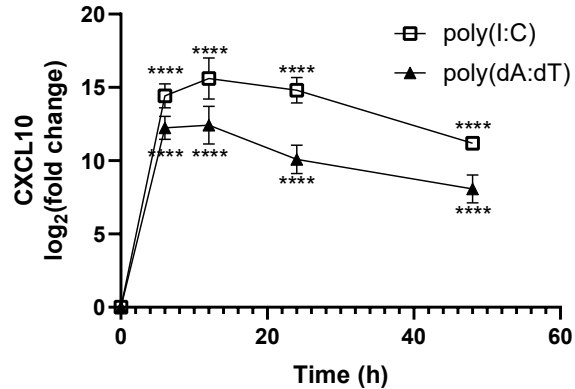

Supplement: S1 Fig — Cells were transfected with 0,666 μg/mL poly(I:C) and 1 μg/mL poly(dA:dT), and samples were collected at 6, 12, 24, 48 hours after transfection. Relative chemokine mRNA expression was determined by the ∆∆Ct method, normalized to GAPDH mRNA expression and fold changes were calculated compared to the expression of the untreated (Control) 0 hours samples. Data are presented as mean of three independent experiments ± SD, white squares representing poly(I:C), black triangles representing poly(dA:dT) treatment. Gene expression differences were analyzed using linear mixed-effects models, for the assessment of time kinetics, expressions were compared to baseline (0 hours) data, *p < 0.05, **p < 0.01, ***p < 0.001, ****p < 0.0001. (PDF) [file pone.0336901.s001.pdf]

**A**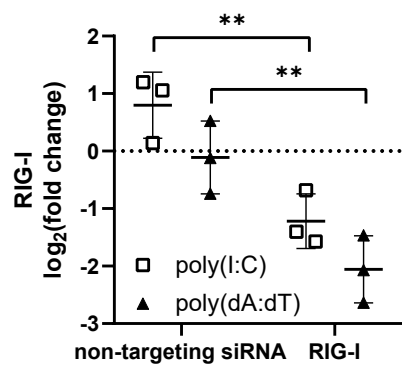**B**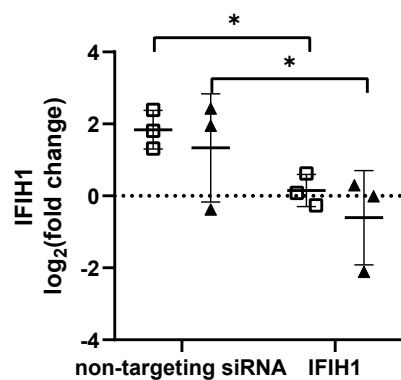**C**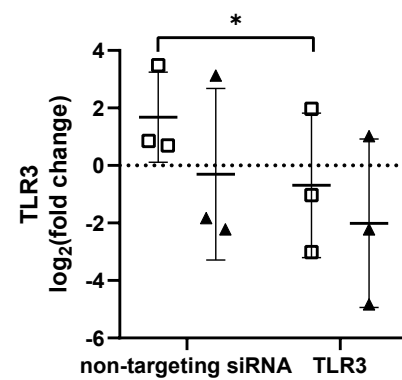**D**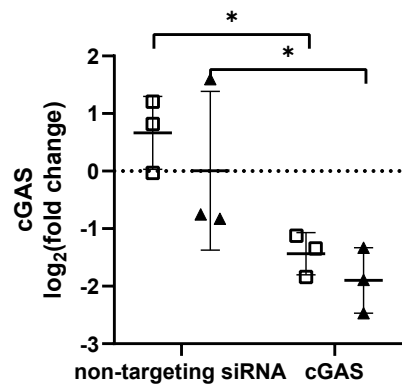**E**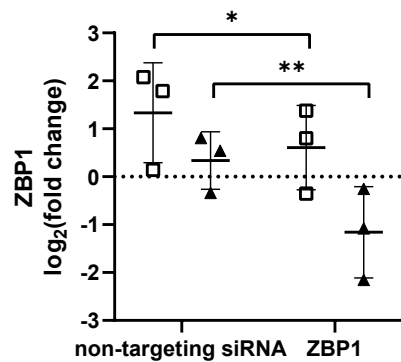**F**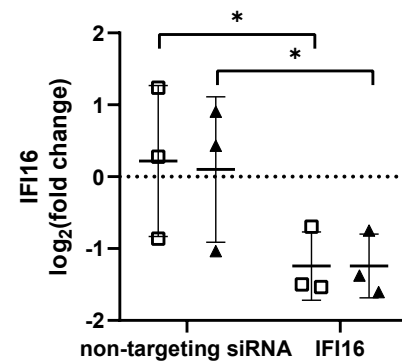**G**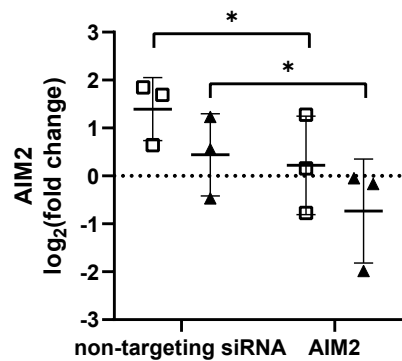**H**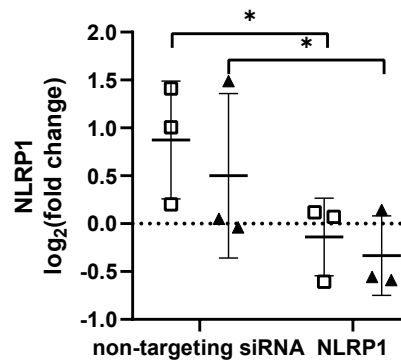**I**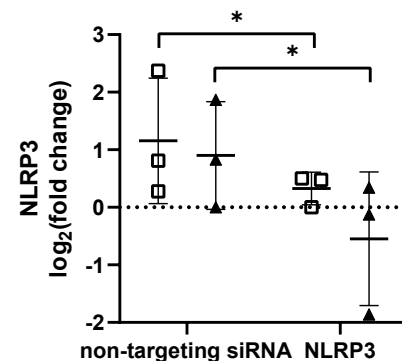

Supplement: S2 Fig — Genexpression of each nucleic acid receptor was silenced using siRNA-mediated gene silencing. Relative mRNA expression of each silenced receptor RIG-I (A), IFIH1 (B), TLR3 (C), cGAS (D), ZBP1 (E), IFI16 (F), AIM2 (G), NLRP1 (H), NLRP3 (I) was determined by the ∆∆Ct method, normalized to GAPDH mRNA expression and compared to the expression of the mock-transfected control samples. Data are presented as mean ± SD with individual datapoints, white squares representing poly(I:C), black triangles representing poly(dA:dT) treatment, n = 3. Gene expression differences were analyzed using linear mixed-effects models with donor as a random effect to account for inter-individual variability, each siRNA was compared to the non-targeting control siRNA within each stimulus condition, * p < 0.05, **p < 0.01. (PDF) [file pone.0336901.s002.pdf]

**A**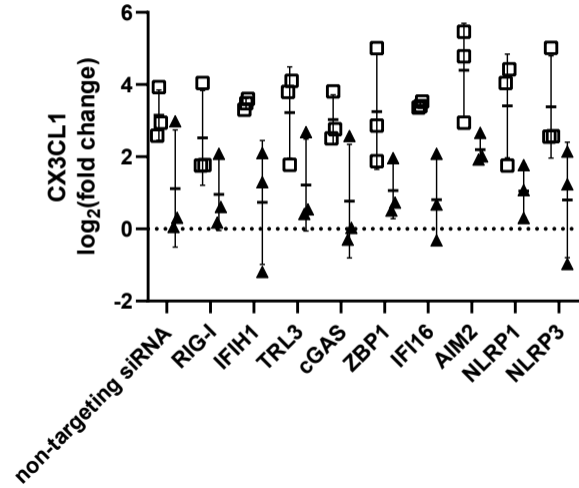**B**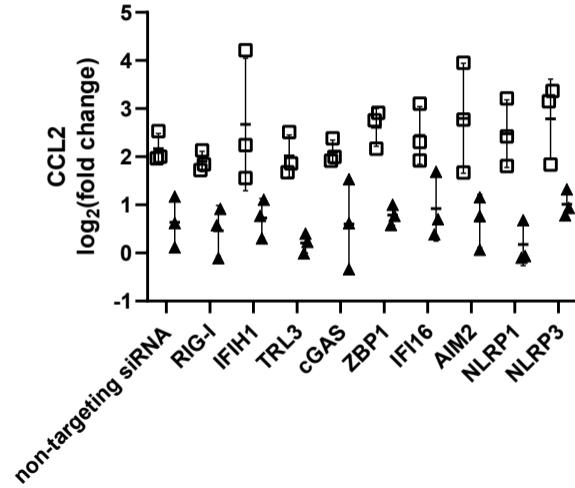**C**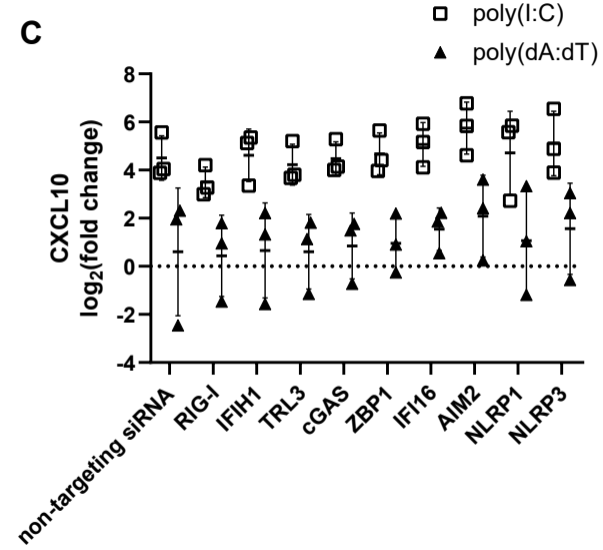

Supplement: S3 Fig — Expression of pattern recognition receptors were silenced with siRNA mediated inhibiton and the cells were transfected with 0,666 μg/mL poly(I:C) and 1 μg/mL poly(dA:dT). Relative fractalkine (A), CCL2 (B) and CXCL10 (C) mRNA expression was determined by the ∆∆Ct method, normalized to GAPDH mRNA expression and compared to the expression of the mock-transfected control samples. Data are presented as mean ± SD with individual datapoints, white squares representing poly(I:C), black triangles representing poly(dA:dT) treatment, n = 3. Gene expression differences were analyzed using linear mixed-effects models with donor as a random effect to account for inter-individual variability, each siRNA was compared to the non-targeting control siRNA within each stimulus condition. (PDF) [file pone.0336901.s003.pdf]

Fractalkine

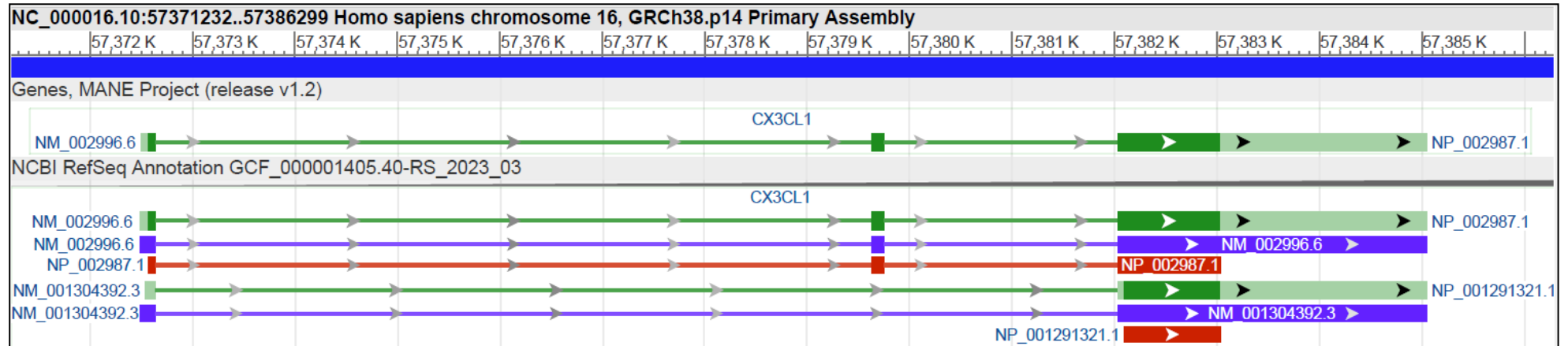

CCL2

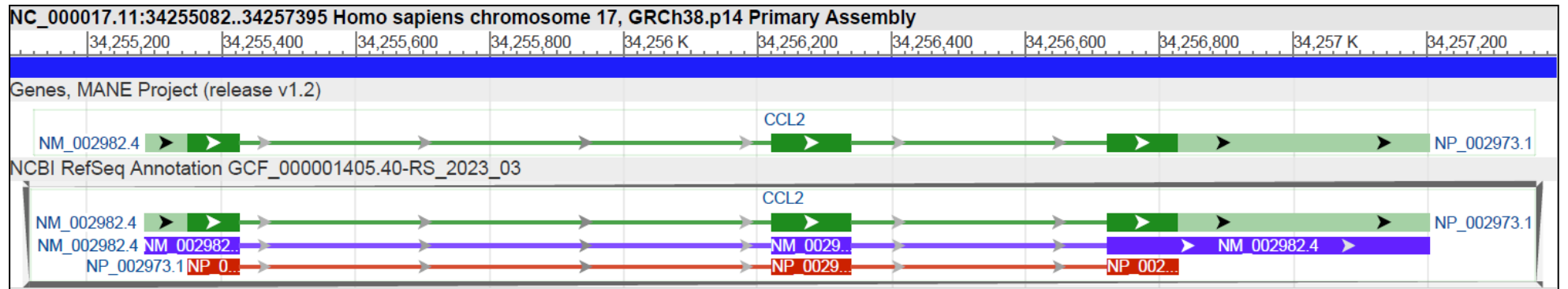

CXCL10

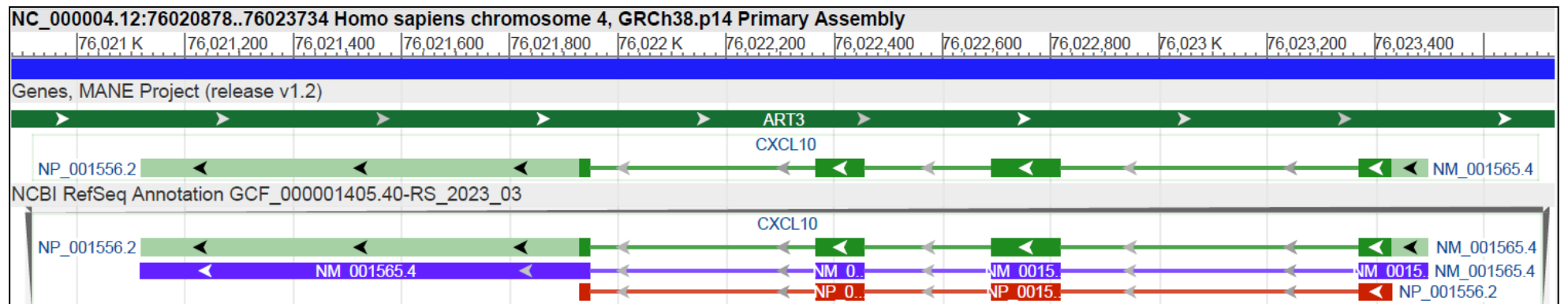

Supplement: S4 Fig — Accessed on 10.10.2023. (PDF) [file pone.0336901.s004.pdf]

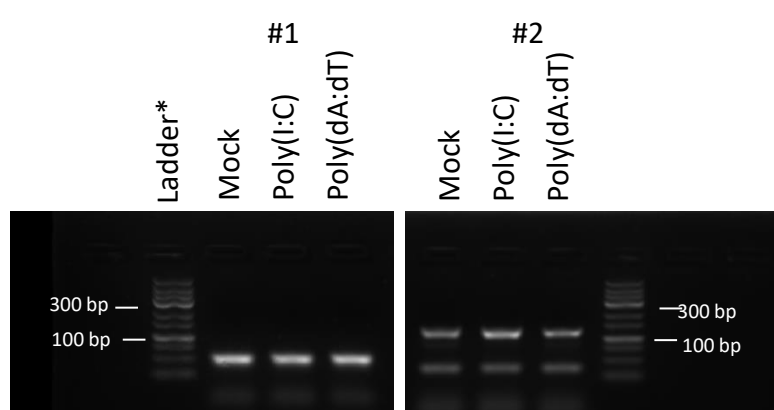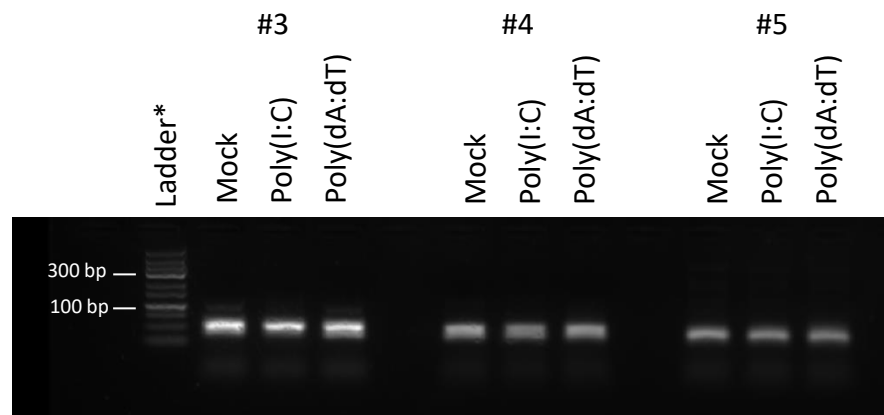

\*Gene Rule Low Range DNA Ladder 25-700bp, Thermo Scientific

Supplement: S5 Fig — To determine splice variant of fractalkine cDNA from keratinocytes transfected by poly(I:C) or poly(dA:dT) was used in PCR reactions using the DreamTaq Green DNA Polymerase (Thermo Fischer Scientific) according to the manufacturer’s instructions. PCR products underwent electrophoresis on 4% agarose gel, and were visualized on the Omega Lum G Chemidoc Imaging System (Aplegen, Inc, Pleasanton, CA, USA). Numbers correspond to the respectieve PCR-primer pairs listed in S1 Table. Products were cut out, isolated by the Nucleospin Gel and PCR Cleanup kit (Macherey Nagel) and subjected to Sanger sequencing. (PDF) [file pone.0336901.s005.pdf]
