# Supplementary material for: Nucleic acid-induced chemokine expression in keratinocytes: Implications for skin inflammation
Source: PLoS One. 2025 Nov 20;20(11):e0336901. doi: 10.1371/journal.pone.0336901 (PMC12633943; doi:10.1371/journal.pone.0336901)
Supplement: S1 Table — (PDF) [file pone.0336901.s006.pdf]

S1 Table

			Expected Product Size		Products
		5'-3' sequence	NM_002996	NM_001304392	
1	FWD	ccaccttctgccatctgac	64 kb	-	NM_002996
	REV	atgttgcatttcgtcacacc			
2	FWD	gcggcaaacgcgcaatca	128 kb	-	NM_002996
	REV	gccgccatttcgagttag			
3	FWD	ctggctgc ttggagacga gac	-	156 kb	no specific product
	REV	gtcctgggcttcacctcgccg			
4	FWD	gctggctgcttggag	-	157 kb	no specific product
	REV	gtcctgggcttcacctcgccg			
5	FWD	gtcctgctggctgcttg	-	162 kb	no specific product
	REV	gtcctgggcttcacctcgccg			
